# Optimizing biochar, vermicompost, and duckweed amendments to mitigate arsenic uptake and accumulation in rice (*Oryza sativa* L.) cultivated on arsenic-contaminated soil

**DOI:** 10.1186/s12870-024-05219-w

**Published:** 2024-06-13

**Authors:** Rana Roy, Akram Hossain, Md. Omar Sharif, Mitali Das, Tanwne Sarker

**Affiliations:** 1 Department of Agroforestry & Environmental Science, Sylhet Agricultural University, Sylhet, 3100 Bangladesh; 2 Institute of Plant Nutrition and Soil Science, Christian-Albrechts-Universität zu Kiel, 24118 Kiel, Germany; 3 Department of Sociology and Rural Development, Khulna Agricultural University, Khulna, 9100 Bangladesh

**Keywords:** Arsenic-contamination, Biochar, Duckweed, *Oryza sativa*, Vermicompost

## Abstract

The accumulation of arsenic (As) in rice (*Oryza sativa* L.) grain poses a significant health concern in Bangladesh. To address this, we investigated the efficacy of various organic amendments and phytoremediation techniques in reducing As buildup in *O. sativa*. We evaluated the impact of five doses of biochar (BC; BC_0.1_: 0.1%, BC_0.28_: 0.28%, BC_0.55_: 0.55%, BC_0.82_: 0.82% and BC_1.0_: 1.0%, w/w), vermicompost (VC; VC_1.0_: 1.0%, VC_1.8_: 1.8%, VC_3.0_: 3.0%, VC_4.2_: 4.2% and VC_5.0_: 5.0%, w/w), and floating duckweed (DW; DW_100_: 100, DW_160_: 160, DW_250_: 250, DW_340_: 340 and DW_400_: 400 g m^− 2^) on *O. sativa* cultivated in As-contaminated soil. Employing a three-factor five-level central composite design and response surface methodology (RSM), we optimized the application rates of BC-VC-DW. Our findings revealed that As contamination in the soil negatively impacted *O. sativa* growth. However, the addition of BC, VC, and DW significantly enhanced plant morphological parameters, SPAD value, and grain yield per pot. Notably, a combination of moderate BC-DW and high VC (BC_0.55_VC_5_DW_250_) increased grain yield by 44.4% compared to the control (BC_0_VC_0_DW_0_). As contamination increased root, straw, and grain As levels, and oxidative stress in *O. sativa* leaves. However, treatment BC_0.82_VC_4.2_DW_340_ significantly reduced grain As (G-As) by 56%, leaf hydrogen peroxide by 71%, and malondialdehyde by 50% compared to the control. Lower doses of BC-VC-DW (BC_0.28_VC_1.8_DW_160_) increased antioxidant enzyme activities, while moderate to high doses resulted in a decline in these activities. Bioconcentration and translocation factors below 1 indicated limited As uptake and translocation in plant tissues. Through RSM optimization, we determined that optimal doses of BC (0.76%), VC (4.62%), and DW (290.0 g m^− 2^) could maximize grain yield (32.96 g pot^− 1^, 44% higher than control) and minimize G-As content (0.189 mg kg^− 1^, 54% lower than control). These findings underscore effective strategies for enhancing yield and reducing As accumulation in grains from contaminated areas, thereby ensuring agricultural productivity, human health, and long-term sustainability. Overall, our study contributes to safer food production and improved public health in As-affected regions.

**Supplementary Information:**

The online version contains supplementary material available at 10.1186/s12870-024-05219-w.

## Introduction

Arsenic (As), a metalloid, is known for its toxic nature and can naturally occur in soils. Its carcinogenic and poisonous properties make soil contamination with As a serious risk to the environment, agriculture, and human health [1, 2]. The utilization of As-contaminated groundwater for irrigation and agricultural purposes, as well as the use of agrochemicals and the disposal of mining waste, have contributed to an increase in As concentration in agricultural soils [3]. This situation becomes more problematic when arsenate enters the food chain, affecting a wide range of plants and animals. High levels of As have been detected in rice (*Oryza sativa* L.) grains irrigated with As-rich groundwater [4]. The accumulation of contaminants in *O. sativa* grains and their consumption pose significant health risks [5, 6]. This issue is particularly severe for the people of Bangladesh, where per capita consumption of *O. sativa* is 400 g per day, constituting over 80% of their caloric intake.

In addition to its detrimental effects on human health, the adverse effects of As on plant growth and development are well documented [7]. The As, even in small amounts, hinders the growth of plants and interferes with crucial activities such as photosynthesis and the absorption of nutrients [8]. The signs of chlorosis and necrosis are easily observed, indicating that As disrupts the process of chlorophyll production and photosynthesis. The As competes with important nutrients such as phosphate, resulting in decreased absorption and translocation [9]. Additionally, oxidative stress induced by reactive oxygen species due to As exposure, damages cellular components [10]. Morphologically, plants exposed to As show reduced leaf areas, stunted growth, and abnormal root development. Furthermore, reproductive processes are negatively impacted, resulting in decreased rates of seed germination and pollen viability [11]. A recent study conducted by Muehe et al. [12] anticipated that the simultaneous occurrence of soil pollution with As and future climate change would lead to a 39% reduction in crop output and a twofold increase in As levels in *O. sativa* grains. Hence, understanding the response of *O. sativa* to elevated levels of As can facilitate the development of effective strategies to alleviate As toxicity on the growth, productivity, and quality of *O. sativa* grains. Ensuring sustainable *O. sativa* production is imperative in the face of the ongoing climate issue.

To reduce As accumulation in *O. sativa* grain, numerous interventions have been investigated, including organic and inorganic amendments, phytoremediation techniques, and emerging technologies such as seed priming and nanotechnology [13–16]. Seed priming is a technique that includes soaking seeds before planting them in order to improve their ability to germinate and grow into seedlings, especially in challenging environments. This process has the potential to decrease the absorption of As by the seeds [16]. In similar ways, nanotechnology presents new prospects for delivering detoxifying agents to plants specifically, hence reducing the buildup of As in grains [13]. Biochar (BC), an advanced substance with nanoscale capabilities, can also be classified as part of this technological spectrum. Recent research has discovered that BC can effectively reduce the bioavailability and bioaccumulation of As and other heavy metals [17]. This is accomplished by a variety of physicochemical mechanisms, including adsorption, precipitation, and complexation [18, 19]. Additionally, the use of BC can also improve soil physicochemical properties and reduce phytotoxicity, leading to either maintained or increased crop yields [20, 21]. By combining these novel approaches with conventional methodologies, we can provide complete solutions to reduce the toxicity of As in crops such as *O. sativa*. Like BC, vermicomposting is also an eco-friendly way to improve soil physicochemical and biological qualities, as well as crop productivity. Vermicompost (VC) can efficiently retain toxic metals from soil solutions and restrict the transportation of As in *O. sativa* [22]. Furthermore, phytoremediation is receiving increasing attention as a viable alternative for As remediation that is both cost-effective and ecologically sustainable. Duckweed (DW) (*Lemna minor* L.) has the greatest capacity to acquire As from contaminated water, and its prevalence in flooded rice fields is widespread in Bangladesh [23, 24].

Numerous studies have investigated the individual effects of BC, VC, and DW on *O. sativa* cultivated in As-contaminated soil, revealing their potential to mitigate As buildup in the seedlings [23, 25, 26]. However, no study has examined how these amendments work together to minimize the negative effects of As in *O. sativa*, and their ideal application rate to reduce As buildup is unknown. In Bangladesh, where BC, VC, and DW are not only economically accessible but also abundantly available, it is crucial to investigate practical solutions that utilize these cost-effective and locally abundant resources to effectively mitigate the adverse impacts of As on *O. sativa*. Given that *O. sativa* is a crop of great worldwide importance and a staple in Bangladesh, it is crucial to address the issue of As contamination in *O. sativa* in order to safeguard food security and public health.

Considering the above facts, we set our objectives (i) to evaluate the efficiency of combined applications of BC, VC, and DW on morpho-physiological and biochemical growth attributes of *O. sativa* in As-contaminated soil and (ii) to identify the best BC-VC-DW combination that can enhance *O. sativa* grain yield (GY) while reducing the As content in the grains (G-As). Here, we hypothesize that the strategic use of BC, VC, and DW will not only improve the GY but also reduce the G-As content in the *O. sativa* in As-contaminated soil. The findings of the study could be an eco-friendly and low-cost technique to reduce As content from the *O. sativa* grain and ensure food security applicable in Bangladesh and worldwide.

## Methodology

### Pot experiment

The pot experiment was conducted in a plastic shed at Sylhet Agricultural University, Bangladesh (24°54′33.12″ N, 91°54′7.2″ E) (Fig. S1). Soil samples were collected from the nearby area at a depth ranging from 0 to 15 cm. These soil samples were then placed in a net house and left to dry naturally. Prior to potting, soil was ground manually and mixed well, and physical and chemical properties were determined. The properties of soil, BC, and VC before starting the experiment were presented in Table 1.

**Table 1:** Properties of soil samples, biochar, and vermicompost before application of fertilizer

Materials	pH	OM (%)	OC (%)	Total-*N* (%)	Available *P*	Exchangeable K	Total As (mg kg^− 1^)
Soil	5.2	2.58	1.5	0.129	23.1 mg kg^− 1^	0.28 (meq 100 g^− 1^ soil)	0.03
Biochar (BC)	11.01	-	7.28	0.626	0.81%	0.98%	-
Vermicompost (VC)	7.3	18.30	-	0.72	251.3 mg kg^− 1^	0.73 (meq 100 g^− 1^ soil)	-

The SL-8 H, a high-yielding hybrid variety of *O. sativa* seed, was obtained from the Bangladesh Agricultural Development Corporation (BADC) and utilized as the test crop due to its widespread cultivation in the Sylhet region, Bangladesh. Five kilograms of dry soil were placed into each plastic pot, and varying concentrations of BC and VC were added according to the experimental design. The soil was then flooded with water and left overnight. The following day, sodium arsenate (Na_2_HAsO_4_.7H_2_O) was applied at a concentration of 20 mg kg^− 1^, and the pots were incubated for seven days before *O. sativa* seedlings were transplanted [27–29]. The soil was amended with the recommended doses of urea (120 ppm), triple superphosphate (25 ppm), potassium (40 ppm), sulfur (12.5 ppm), and gypsum (120 ppm). The urea was applied in three equal doses: one-third as a basal dose, and the second and third applications were conducted at 30 days (during the maximum tillering stage) and 70 days (during the panicle initiation stage) after transplantation, respectively. Two 45-day-old *O. sativa* seedlings were transplanted into each pot, and varying concentrations of DW were added. From the time of transplantation until physiological maturity, the water level was maintained at 2–4 cm above the soil level in the pots using As-free tap water. Throughout the growing season, several cross-cultural interventions were implemented.

### Experimental design

The response surface methodology (RSM) was employed to optimize BC, VC, and DW application rates in order to get maximum GY and minimum G-As in *O. sativa*. The optimization process involved the selection of a central composite design (CCD). The dependent or response variables in the study comprised *O. sativa* GY, As content, and a variety of morpho-physiological and biochemical growth indicators, while the independent variables were BC, VC, and DW. We selected five different levels (+ 1.682, + 1, 0, -1, -1.682) of BC, VC, and DW (provided in Table 2 and Fig. S2), and their application rates were chosen based on the previous literature [30–32].

**Table 2:** The independent variables with their coded and actual values

Independent variables	Codes	Coded and actual values				
		-1.682	-1	0	+ 1	+ 1.682
		Lowest dose	Low dose	Moderate dose	High dose	Highest dose
Biochar (BC) (%, w/w)	A	0.1	0.28	0.55	0.82	1.0
Vermicompost (VC) (%, w/w)	B	1.0	1.8	3.0	4.2	5.0
Duckweed (DW) (g m^‒2^)	C	100	160	250	340	400

The number of experimental treatments, as determined by RSM, was 18, including the control (Presented in Table 3). Three replicates of each treatment were conducted, resulting in a total of 54 pots. These pots were randomly arranged in the plastic shed.

A second-order polynomial model was used to fit the experimental data:

1 $${\rm{Y = {\beta _0}{\rm{ + }}{\beta _1}A + {\beta _2}B + {\beta _3}C + {\beta _{12}}AB}} + {\beta _{13}}AC + {\beta _{23}}BC + {\beta _{11}}{A^2} + {\beta _{22}}{B^2} + {\beta _{33}}{C^2}$$

Here, the variable Y is used to represent response variables, the constant coefficient is denoted by β_0_, the interpret linear coefficients are denoted by β_1_, β_2_, and β_3_, the interaction coefficients are denoted by β_12_, β_13_, and β_23_, the quadratic coefficients are denoted by β_11_, β_22_, and β_33_, and the coded values of BC, VC, and DW are denoted by the letters A, B, and C respectively.

**Table 3:** Treatment combinations and their experimental layout in the central composite design

Treatments		Coded level of factors			Quantity applied		
		Biochar (BC)	Vermicompost (VC)	Duckweed (DW)	BC (%, w/w)	VC (%, w/w)	DW (g m^‒2^)
1	Control (BC_0_VC_0_DW_0_)	-	-	-	0	0	0
2	BC_0.28_VC_1.8_DW_160_	-1	-1	-1	0.28	1.8	160
3	BC_0.82_VC_1.8_DW_160_	1	-1	-1	0.82	1.8	160
4	BC_0.28_VC_4.2_DW_160_	-1	1	-1	0.28	4.2	160
5	BC_0.82_VC_4.2_DW_160_	1	1	-1	0.82	4.2	160
6	BC_0.28_VC_1.8_DW_340_	-1	-1	1	0.28	1.8	340
7	BC_0.82_VC_1.8_DW_340_	1	-1	1	0.82	1.8	340
8	BC_0.28_VC_4.2_DW_340_	-1	1	1	0.28	4.2	340
9	BC_0.82_VC_4.2_DW_340_	1	1	1	0.82	4.2	340
10	BC_0.1_VC_3_DW_250_	-1.682	0	0	0.10	3.0	250
11	BC_1_VC_3_DW_250_	1.682	0	0	1.00	3.0	250
12	BC_0.55_VC_1_DW_250_	0	-1.682	0	0.55	1.0	250
13	BC_0.55_VC_5_DW_250_	0	1.682	0	0.55	5.0	250
14	BC_0.55_VC_3_DW_100_	0	0	-1.682	0.55	3.0	100
15	BC_0.55_VC_3_DW_400_	0	0	1.682	0.55	3.0	400
16	BC_0.55_VC_3_DW_250_	0	0	0	0.55	3.0	250
17	BC_0.55_VC_3_DW_250_	0	0	0	0.55	3.0	250
18	BC_0.55_VC_3_DW_250_	0	0	0	0.55	3.0	250

*In above, BC_0.28_ means biochar @ 0.28% w/w, VC_1.8_ means vermicompost @ 1.8% w/w, and DW_160_ means duckweed @ 160 g m^‒2^

### Assessment of morpho-physiological and biochemical growth parameters

The growth of *O. sativa* was assessed by measuring various parameters including shoot length (SL, cm), root length (RL, cm), panicle length (PL, cm), grain yield g pot^− 1^ (GY), number of filled grains panicle^− 1^ (NFG), number of unfilled grains panicle^− 1^ (NUG), 1000-grain weight (1000-GW, g), and above-ground biomass (ABG, g), and below-ground biomass (BGB, g). The SL, RL, and PL were measured by a meter scale. The ABG and BGB were determined by subjecting plant samples to a drying process in an oven set at a temperature of 80 °C for 72 h [33]. The chlorophyll content of *O. sativa* was quantified using the SPAD (Soil Plant Analysis Development) method. The assessment was conducted using a portable Minolta chlorophyll meter (SPAD-502, Osaka 590–8551, Japan) [34].

To measure the As content (mg kg^− 1^), root, straw, and grain samples underwent digestion using a mixture of nitric acid (HNO_3_) and perchloric acid (HClO_4_) in order to measure the concentrations of As using a hydride generation atomic absorption spectrophotometer [35].

Hydrogen peroxide (H_2_O_2_, µmol g^‒1^ FW) levels were determined according to Velikova et al. [36]. Leaf tissues (500 mg) were homogenized in the ice bath with 5 ml 0.1% (w/v) trichloroacetic acid (TCA). The homogenate was centrifuged at 10,000 rpm for 15 min and 0.5 ml of the supernatant was added to 0.5 ml 10 mM potassium phosphate buffer (pH 7.0) and 1 ml 1 M Potassium iodide, and the absorbance of the supernatants was read at 390 nm. The malondialdehyde (MDA, µmol g^‒1^ FW) content in leaf samples of *O. sativa* was estimated as described by Roy et al. [37]. Extract (2 mL) was mixed with an equal volume of 0.5% (w/v) 2-thiobarbituric acid (TBA) (TBA, dissolved in 15% TCA), and the mixture was heated for 30 min at 100 °C followed by cooling in an ice bath. The mixture was then centrifuged at 10,000 rpm for 10 min, and the absorbance of the supernatants was read at 450, 532, and 600 nm using Pharmacia Ultra Spec Pro UV/VIS spectrophotometer (Pharmacia, Cambridge, England).

Fresh leaf samples were homogenized in a mortar and pestle (ice-cold conditions) using 8 mL of 50 mM sodium phosphate buffer (pH 7.8) and the homogenates were centrifuged at 10,000 rpm for 20 min at 4 °C. The supernatant was separated and used for the estimation of enzyme activities [38]. The activity of superoxide dismutase (SOD, EC 1.15.1.1, U g^‒1^ FW) was assayed following the method of Roy et al. [37]. In short, the reaction mixture containing 3 mL of phosphate buffer (pH 7.8), 0.6 mL of 130 mM methionine buffer, 0.6 mL of 750 µM nitroblue tetrazolium buffer, 0.6 mL of 100 µM EDTA-Na buffer and 0.6 mL of 20 µM riboflavin was mixed with 0.2 mL of enzyme extract. The photoreduction of nitroblue tetrazolium was determined at 560 nm. The catalase (CAT, EC 1.11.1.6, U min^‒1^ g^‒1^ FW) activity was measured based on the method of Beers and Sizer [39]. In brief, 100 µL enzyme extract was mixed with a reaction mixture (2.6 mL) containing 100 mM phosphate buffer (pH 7.0) and 20 mM H_2_O_2_. The reduction in H_2_O_2_ was monitored at 240 nm. Enzyme activity was expressed as CAT U min^‒1^ g^‒1^ FW. Ascorbate peroxidase (APX) activity was determined by measuring the reduction of ascorbic acid [40]. Briefly, 0.1 mL of enzyme extract was mixed with 2.9 mL of 50 mM phosphate buffer (pH 7.0) having 0.5 mM ascorbic acid and 0.1 mM H_2_O_2_. The reduction of ascorbic acid was obtained by recording the decrease of absorbance at 290 nm.

The Bioconcentration factor (BCF) and translocation factor (TF) were calculated according to Sikdar et al. [41]. The BCF root (BCF-R), BCF straw (BCF-S), BCF grain (BCF-G), TF root-straw (TFr-s), TF root-grain (TFr-g) were calculated as follows: As_root_/As_soil_, As_straw_/As_soil_, As_grain_/As_soil_, As_straw_/As_root_ and As_grain_/As_root_.

### Data analysis

The optimal BC, VC, and DW rates were obtained through the utilization of Design Expert statistical software version 11 (Stat-Ease, USA). This was accomplished by combining an optimization process with the derringer’s desired function approach. Principal component analysis of the several growth traits was carried out using Origin 2018 (OriginLab Inc, USA). A heatmap was generated using the online program package available at https://biit.cs.ut.ee/clustvis.

## Results

**Fig. 1:**
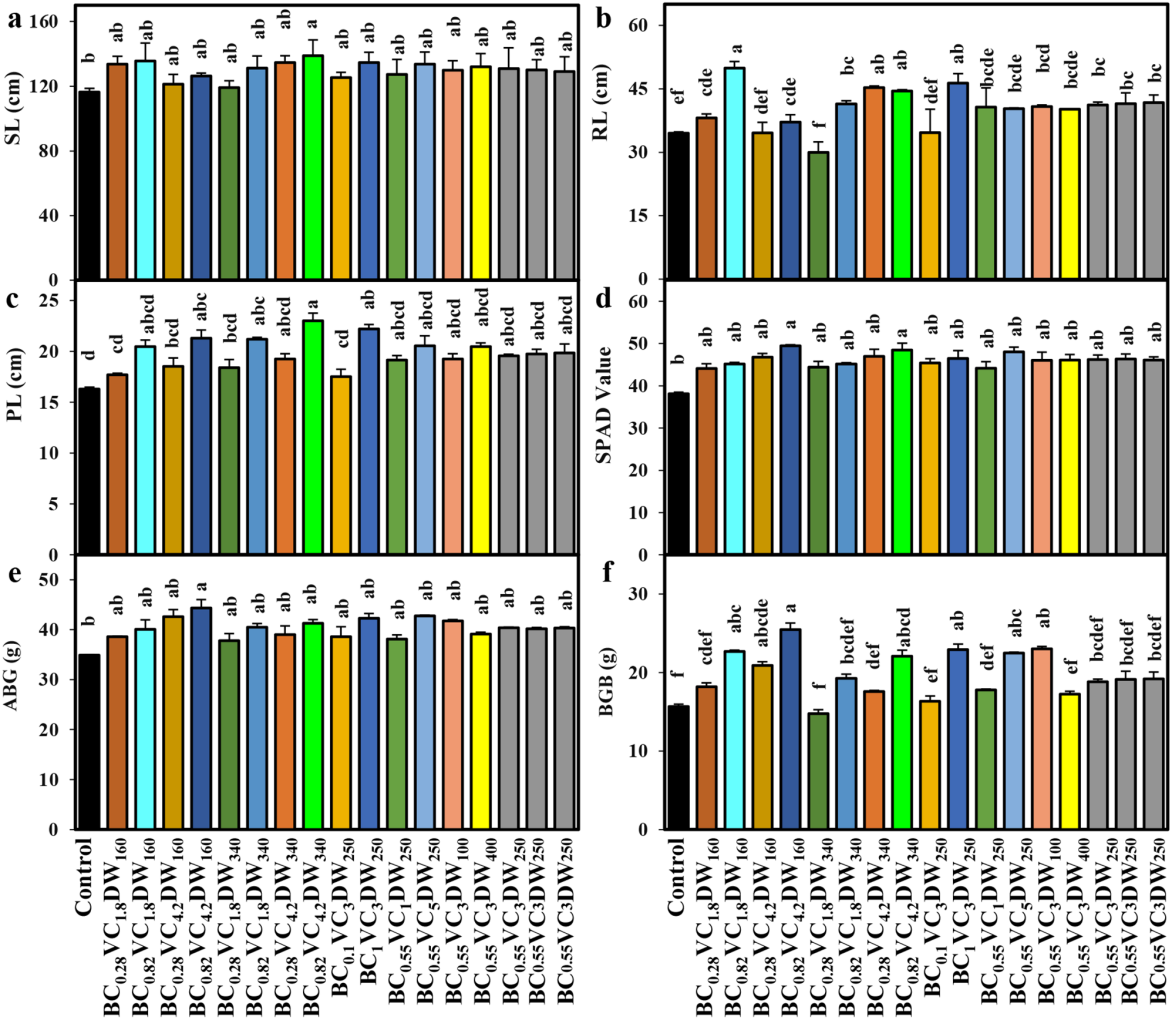
Depicts the effects of various biochar-vermicompost-duckweed (BC-VC-DW) regimes on (**a**) shoot length (SL), (**b**) root length (RL), (**c**) panicle length (PL), (**d**) SPAD, (**e**) above-ground biomass (AGB), and (**f**) below-ground biomass (BGB) of *O. sativa* seedlings. Bars with distinct small letters indicate significant differences at *p <* 0.05. The values represent the mean ± standard error (*n* = 3)

The addition of a high amount of BC, VC, and DW enhanced the shoot length of *O. sativa* (SL) compared to control. More specifically, the BC_0.82_VC_4.2_DW_340_ treatment significantly (*p* < 0.05) increased SL by 19.4% compared to control (Fig. 1a and Fig. S3). The use of BC, VC, and DW in various treatments significantly increased *O. sativa* root length (RL) by 0.2‒44.5% compared to control seedlings (except BC_0.28_VC_1.8_DW_340_) (Fig. 1b). In comparison with control, treatments BC_0.82_VC_4.2_DW_160_, BC_0.82_VC_1.8_DW_340_, BC_0.82_VC_4.2_DW_340_ and BC_1_VC_3_DW_250_ significantly (*p* < 0.05) increased panicle length (PL) by 30.8, 30.1, 41.3 and 36.3%, respectively (Fig. 1c). The application of BC, VC, and DW had a notable impact on the SPAD value, resulting in an increase of 15.6‒29.6% compared to the control treatment (Fig. 1d). The addition of high BC-VC and low DW (BC_0.82_VC_4.2_DW_160_) significantly (*p* < 0.05) increased above-ground biomass (AGB) by 27% compared to control (Fig. 1e). The addition of BC, VC, and DW considerably boosted below-ground biomass (BGB) in *O. sativa* seedlings, rising by 4.3‒62.6% compared to control seedlings (except for BC_0.28_VC_1.8_DW_340_) (Fig. 1f).

**Fig. 2:**
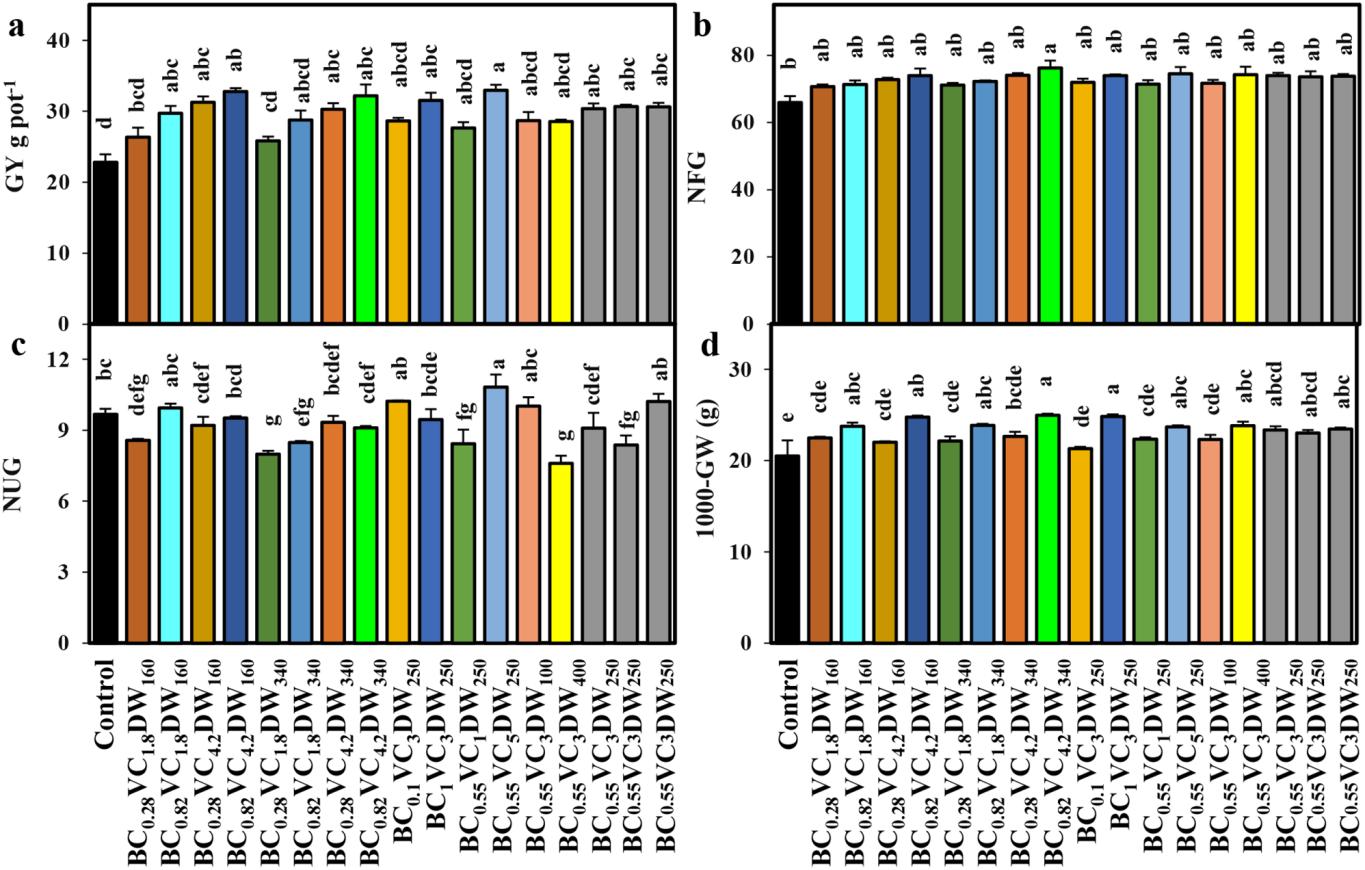
Depicts the effects of various biochar-vermicompost-duckweed (BC-VC-DW) regimes on (**a**) grain yield pot^− 1^, (**b**) number of filled grain panicle^− 1^, (**c**) number of unfilled grain panicle^− 1^ and (**d**) 1000 grain weight of *O. sativa* seedlings. Bars with distinct small letters indicate significant differences at *p <* 0.05. The values represent the mean ± standard error (*n* = 3)

We observed that the addition of BC, VC, and DW significantly increased *O. sativa* grain yield (GY) pot^-1^, with BC_0.55_VC_5_DW_250_ showing the highest increase, measuring 44.4% higher than the control seedlings (Fig. 2a). The treatment BC_0.82_VC_4.2_DW_340_ significantly (*p* < 0.05) increased the number of filled grain panicle^-1^ (NFG) by 15.7% compared to control, while BC_0.55_VC_3_DW_400_ displayed the lowest number (7.6) of unfilled grain panicle^-1^ (NUG) (Fig. 2b-c). Seedlings exposed to BC_0.82_VC_4.2_DW_160_, BC_0.82_VC_1.8_DW_340_, BC_0.82_VC_4.2_DW_340_ and BC_1_VC_3_DC_250_ presented a significant (*p* < 0.05) enhancement in 1000-grain weight (1000-GW), which were 20.8, 16.2, 21.7 and 21.0% higher, respectively, than the control (Fig. 2d).

**Fig. 3:**
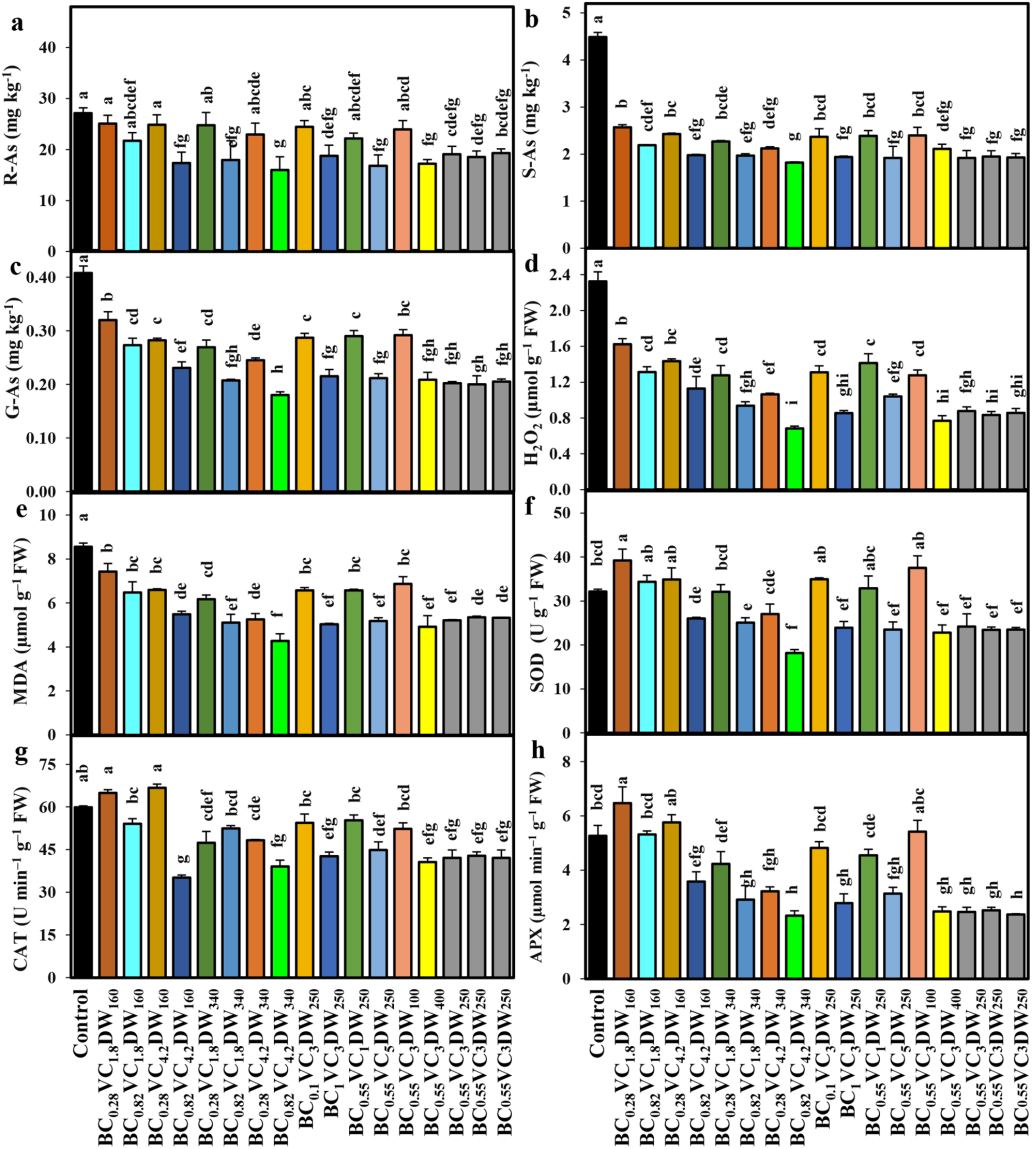
Depicts the effects of various biochar-vermicompost-duckweed (BC-VC-DW) regimes on the contents of (**a**) root-As (R-As), (**b**) straw-As (S-As), (**c**) grain-As (G-As), (**d**) hydrogen peroxide (H_2_O_2_), (**e**) malondialdehyde (MDA), and activities of (**f**) superoxide dismutase (SOD), (**g**) catalase (CAT), and (**h**) ascorbate peroxidase (APX) in *O. sativa* leaves. Bars with distinct small letters indicate significant differences at *p <* 0.05. The values represent the mean ± standard error (*n* = 3)

The As content in *O. sativa* root (R-As), straw (S-As), and grain (G-As) was significantly reduced in all treatments when compared with control. For example, under treatment BC_0.82_VC_4.2_DW_340 _*O. sativa* exhibited remarkable declines in R-As by 41%, S-As by 60%, and G-As content by 56%, relative to control (Fig. 3a-c). All combinations of BC, VC, and DW treatments caused a significant (*p* < 0.05) decline in H_2_O_2_ and MDA contents; however, the plants that were exposed to high (BC_0.82_VC_4.2_DW_340_) and moderate levels (BC_0.55_VC_3.0_DW_250_) of BC, VC, and DW showed a greater decline of H_2_O_2_ and MDA contents over the control (Fig. 3d and e). When *O. sativa* seedlings were subjected to treatments BC_0.28_VC_1.8_DW_160_, BC_0.28_VC_4.2_DW_160_, and BC_0.55_VC_3_DW_100_, their levels of SOD, CAT, and APX dramatically increased in their leaves compared to the control group; all other treatments caused a notable decline in these activities (Fig. 3f-h).

Our results indicate a decrease in the BCF-R, BCF-S, BCF-G, TFr-s, and TFr-g values with the application of BC, VC, and DW. A remarkable decline in BCF-R (19‒47%), BCF-S (43‒60%), BCF-G (22‒56%), TFr-s (26‒44%), and TFr-g (12‒29%) was observed across treatments compared to the control (Table 4). Furthermore, all BCF and TF values, except BCF-R, are lower than 1 (Table 4). We also observed that soil pH increased by 1.72–33.1% across the various treatments compared to the control (Table 4).

**Table 4:** Effects of various biochar-vermicompost-duckweed (BC-VC-DW) regimes on the Bioconcentration Factor (BCF), Translocation Factor (TF) of *O. sativa* and soil pH. Different letters (a, b, c, etc.) indicate significant differences at *P* < 0.05, according to LSD test (*n* = 3). BCF root (BCF-R), BCF straw (BCF-S), BCF grain (BCF-G), TF root-straw (TFr-s) and TF root-grain (TFr-g)

Treatments	BCF-*R*	BCF-S	BCF-G	TFr-s	TFr-g	Soil pH
CK (BC_0_VC_0_DW_0_)	1.506 ± 0.04a	0.225 ± 0.005a	0.02 ± 0.001a	0.171 ± 0.01a	0.015 ± 0.001a	5.23 ± 0.12b
BC_0.28_VC_1.8_DW_160_	1.255 ± 0.02b	0.129 ± 0.003b	0.016 ± 0.001b	0.102 ± 0.01b	0.013 ± 0.001ab	5.52 ± 0.09ab
BC_0.82_VC_1.8_DW_160_	1.086 ± 0.02d	0.11 ± 0.002def	0.014 ± 0.001bcde	0.114 ± 0.01b	0.013 ± 0.001ab	6.38 ± 0.06ab
BC_0.28_VC_4.2_DW_160_	1.242 ± 0.02b	0.122 ± 0.003bc	0.014 ± 0.001bcd	0.101 ± 0.01b	0.011 ± 0.001b	5.87 ± 0.07ab
BC_0.82_VC_4.2_DW_160_	0.868 ± 0.02efg	0.099 ± 0.001fgh	0.012 ± 0.001cdefg	0.123 ± 0.01b	0.013 ± 0.001ab	6.71 ± 0.14ab
BC_0.28_VC_1.8_DW_340_	1.238 ± 0.02b	0.114 ± 0.003cde	0.013 ± 0.001bcdef	0.098 ± 0.01b	0.011 ± 0.001b	5.45 ± 0.14ab
BC_0.82_VC_1.8_DW_340_	0.896 ± 0.01efg	0.099 ± 0.001gh	0.01 ± 0.001efg	0.1 ± 0.02b	0.012 ± 0.001ab	6.46 ± 0.09ab
BC_0.28_VC_4.2_DW_340_	1.147 ± 0.02bcd	0.106 ± 0.002efg	0.012 ± 0.001cdefg	0.11 ± 0.01b	0.011 ± 0.001b	5.81 ± 0.13ab
BC_0.82_VC_4.2_DW_340_	0.8 ± 0.01 g	0.091 ± 0.002 h	0.009 ± 0.001 g	0.1 ± 0.02b	0.011 ± 0.001b	6.54 ± 0.09ab
BC_0.1_VC_3_DW_250_	1.223 ± 0.02bc	0.119 ± 0.001bcd	0.014 ± 0.001bc	0.114 ± 0.01b	0.012 ± 0.001ab	5.32 ± 0.13ab
BC_1_VC_3_DW_250_	0.937 ± 0.01ef	0.097 ± 0.002gh	0.011 ± 0.002defg	0.108 ± 0.01b	0.011 ± 0.002b	6.96 ± 0.17a
BC_0.55_VC_1_DW_250_	1.109 ± 0.03 cd	0.12 ± 0.001bcd	0.015 ± 0.001bc	0.092 ± 0.01b	0.013 ± 0.001ab	6.08 ± 0.12ab
BC_0.55_VC_5_DW_250_	0.839 ± 0.02 fg	0.096 ± 0.002gh	0.011 ± 0.002efg	0.105 ± 0.01b	0.013 ± 0.002ab	6.37 ± 0.15ab
BC_0.55_VC_3_DW_100_	1.198 ± 0.01bcd	0.12 ± 0.005bcd	0.015 ± 0.001bc	0.092 ± 0.02b	0.012 ± 0.001ab	6.27 ± 0.11ab
BC_0.55_VC_3_DW_400_	0.86 ± 0.02efg	0.106 ± 0.004efg	0.01 ± 0.001efg	0.101 ± 0.01b	0.012 ± 0.001ab	6.21 ± 0.12ab
BC_0.55_VC_3_DW_250_	0.953 ± 0.03ef	0.096 ± 0.006gh	0.01 ± 0.001 fg	0.104 ± 0.01b	0.011 ± 0.001b	6.22 ± 0.13ab
BC_0.55_VC_3_DW_250_	0.927 ± 0.03ef	0.098 ± 0.007gh	0.01 ± 0.001 g	0.104 ± 0.01b	0.011 ± 0.001b	6.24 ± 0.11ab
BC_0.55_VC_3_DW_250_	0.965 ± 0.03e	0.097 ± 0.008gh	0.01 ± 0.002efg	0.107 ± 0.01b	0.011 ± 0.002b	6.26 ± 0.12ab

### Interactive effects of BC, VC, and DW on GY and G-As of *O. sativa* using RSM

**Fig. 4:**
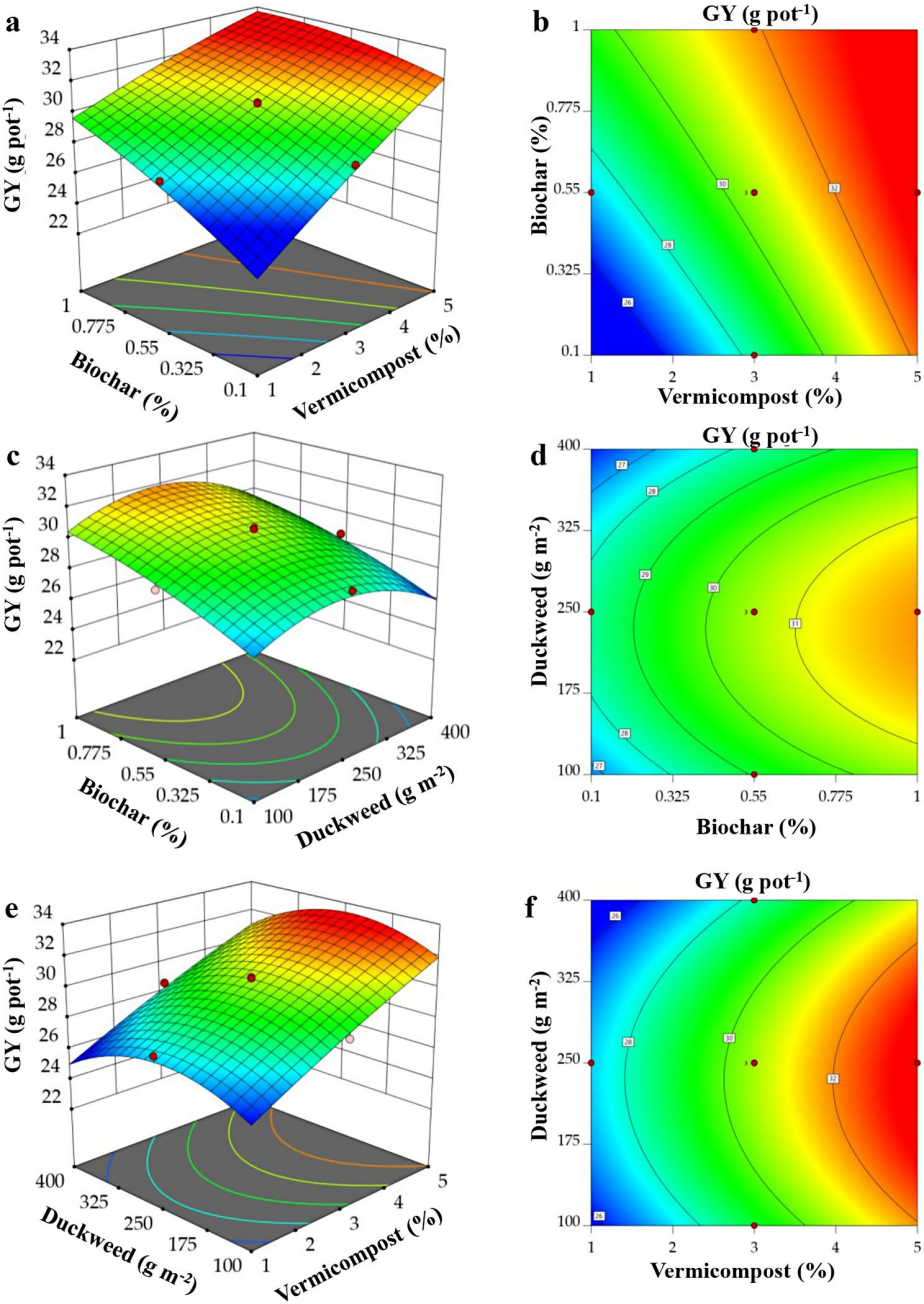
Response surface plots displaying the interaction effects of (**a**-**b**) biochar and vermicompost, (**c**-**d**) biochar and duckweed, (**e**-**f**) duckweed and vermicompost on *O. sativa* grain yield (GY) g pot^− 1^

**Fig. 5:**
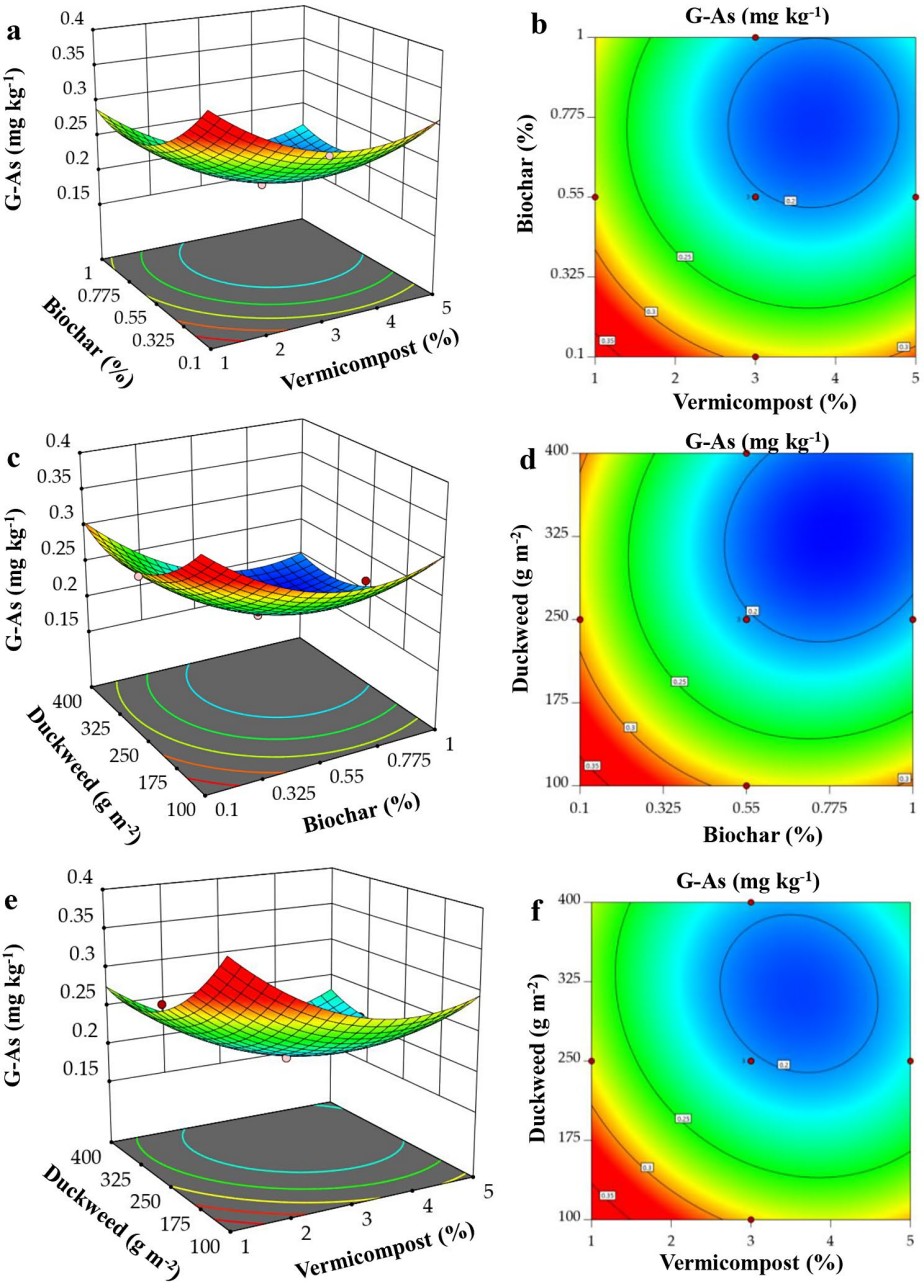
Response surface plots displaying the interaction effects of (**a**-**b**) vermicompost and biochar, (**c**-**d**) duckweed and biochar, (**e**-**f**) duckweed and vermicompost on grain As content (G-As)

Utilizing RSM, 3D surface plots were made to look into how BC, VC, and DW interact with each other to affect the GY and G-As of *O. sativa*. The relationship between the two variables (x and y) and their impact on GY and G-As in the z-axis is depicted in Figs. 4a–f and 5a‒f. Each figure illustrates the impact of two factors while maintaining the third factor at a central level.

The interactive effect of BC, VC, and DW over GY displayed that under As contaminated soil, the GY of *O. sativa* increased linearly with the mutual increase of BC and VC additions (Fig. 4a and b). Interactive effects of DW × BC (Fig. 4c and d) and DW × VC (Fig. 4e and f) showed that the GY initially increased with the increase of DW concentrations up to a certain level (250 g m^‒2^), whereas it started to decline with the increase of DW above this level. However, GY consistently increased with the increase in BC and VC additions, regardless of DW level.

All three factors considerably decreased the G-As content in *O. sativa*. Figure 5a-f showed that G-As content markedly increased with the decrease of BC, VC, and DW doses. The RSM plot demonstrates that G-As content was found to be 0.187 mg kg^-1^ with the addition of VC from 2.6 to 4.7% and BC from 0.55 to 1.0% (Fig. 5a–b). The addition of DW from 230 to 400 g m^-2^ and BC from 0.48 to 1.0% also has a remarkable effect on decreasing the G-As content, which was 0.182 mg kg^-1^ (Fig. 5c–d). Similarly, supplementation of DW from 240 to 390 g m^-2^ and VC from 2.6 to 4.6% resulted in 0.188 mg kg^-1^ As in the *O. sativa* grain (Fig. 5e–f).

The regression coefficients in Table S1 provide the significance and nature of the effects of the factors (BC, VC, DW) on the different growth responses of *O. sativa*. Positive coefficients indicate a positive relationship between the factor and the response variable, while negative coefficients indicate a negative relationship. The positive coefficients for β_1_, β_2_, and β_3_ suggest that the additions of BC, VC, and DW have synergistic effects on the growth traits of *O. sativa*. Specifically, BC and VC additions exhibit synergistic effects on several growth parameters such as SL, RL, PL, SPAD, GY, NFG, NUG, 1000-GW, AGB, and BGB. However, they have antagonistic effects on some other parameters. Similarly, DW additions have synergistic effects on certain growth parameters like SL, RL, PL, NFG, and 1000-GW, but antagonistic effects on other parameters. The coefficients β_12_, β_13_, and β_23_ represent the interaction effects BC × VC, BC × DW, and VC × DW, respectively, on various growth traits of *O. sativa* (Table S1).

### Principal component analysis and heatmap methods

**Fig. 6:**
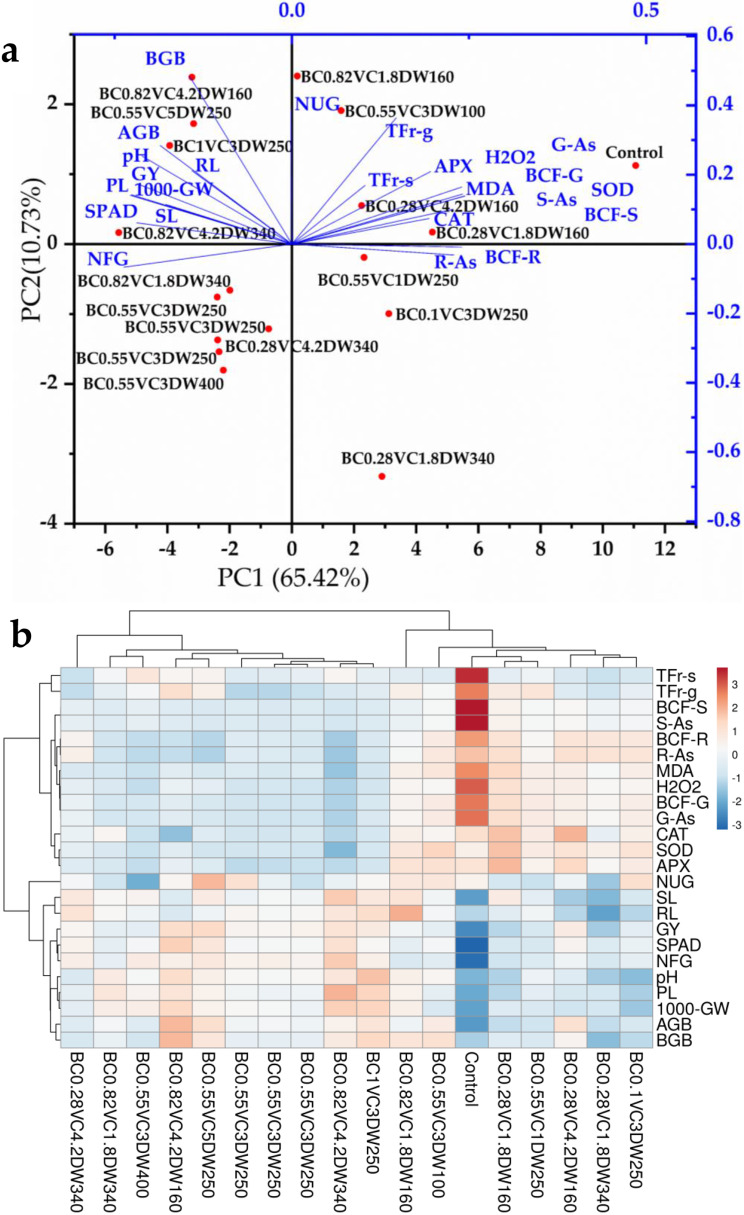
(**a**) Principal component analysis (**a**) and a heatmap (**b**) show how different biochar-vermicompost-duckweed (BC-VC-DW) regimes influence the morphological and physiological growth responses of *O. sativa*

The first seven principal components (PCs) of principal component analysis (PCA) were associated with eigen values above one, and the first two PCs explained 76.15% (PC1 = 65.42% and PC2 = 10.73%) of the total variation (Fig. 6a).

Figure 6a showed that increases in BC and VC doses caused a clear separation of PC1, with the high BC-VC-containing treatments positioned on the left side of the PC1 positively connected with BGB, AGB, pH, GY, RL, PL, 1000-GW, SPAD, SL, and NFG, and negatively correlated with the contents of R-As, S-As, G-As, BCF-R, BCF-G, BCF-S, TFr-s, TFr-g, H_2_O_2_, and MDA, and activities of SOD, CAT, and APX. Whereas the lowest BC and VC-containing treatments were located on the right side of PC1 and demonstrated positive associations with plant As content, ROS, and antioxidant enzyme activities (Fig. 6a).

The heatmap analysis revealed two primary clusters, which corresponded to the BC_0.82_VC_1.8_DW_160_, BC_0.55_VC_3_DW_100_, control, BC_0.28_VC_1.8_DW_160_, BC_0.55_VC_1_DW_250_, BC_0.28_VC_4.2_DW_160_, BC_0.28_VC_1.8_DW_340_ and BC_0.1_VC_3_DW_250_ on the right, while the remaining treatments were located on the left side (Fig. 6b). Treatments with low SL, RL, GY, SPAD, NFG, pH, PL, 1000-GW, AGB, and BGB, and high R-As, S-As, G-As, BCF-R, BCF-G, BCF-S, TFr-s, TFr-g, H_2_O_2_, MDA, and antioxidant enzyme activities clustered on the right. In contrast, the treatments grouped together on the left because they had low levels of As accumulation, H_2_O_2_, MDA contents, antioxidant enzyme activities, and high levels of morphological growth traits (Fig. 6b).

### Fitting the RSM and identification of optimum BC, VC, and DW combination

ANOVA suggested quadratic, 2FI, and linear models for various growth parameters. Table S2 demonstrates that all models had P-values below 0.05 and lack-of-fit F-values greater than 0.05, indicating their statistical validity. Differences between the adjusted R² and predicted R² values were within 0.2 of each other, except for SL and NUG, suggesting a robust correlation between them [42].

**Fig. 7:**
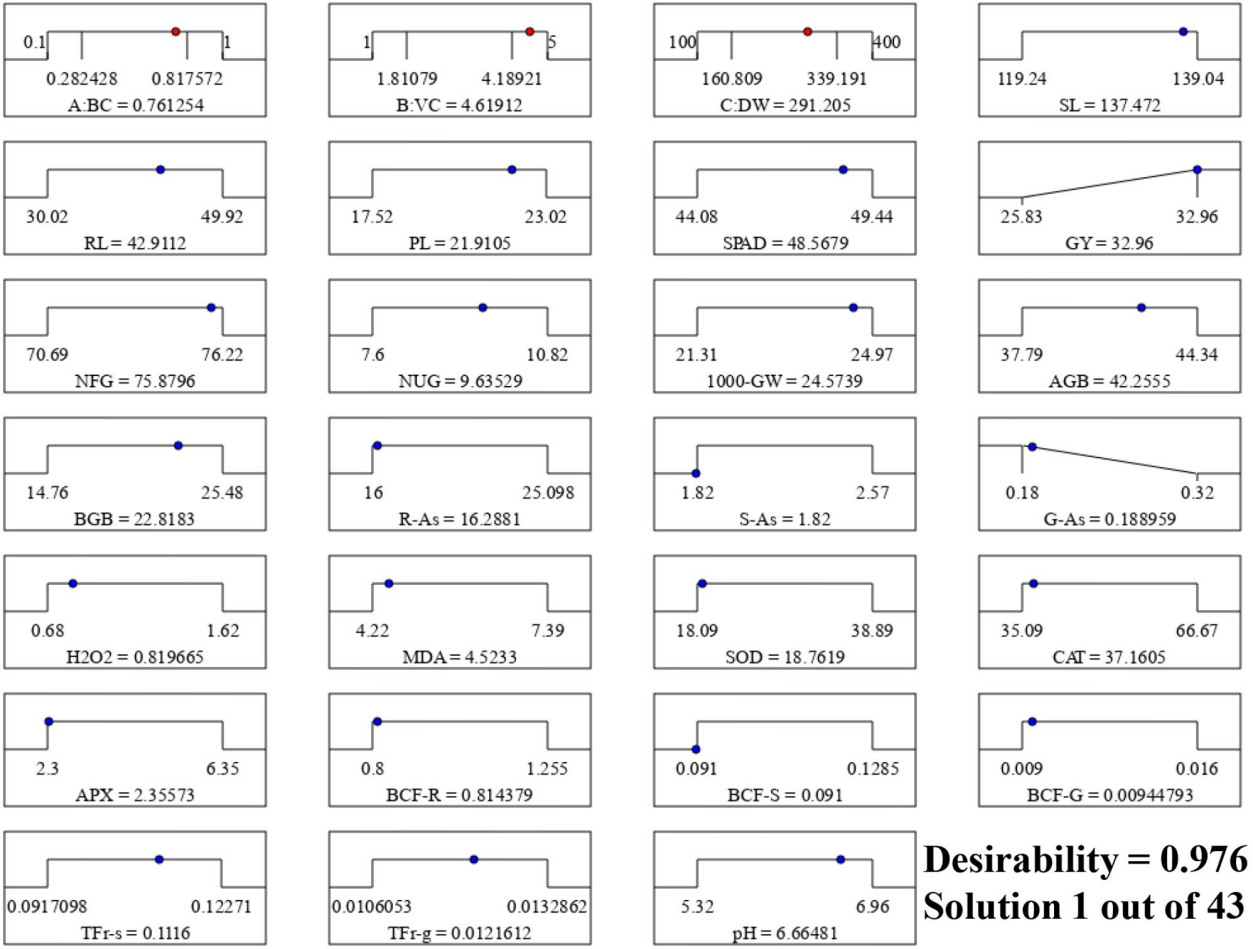
Desirability ramp showing the optimization of biochar (BC), vermicompost (VC), and duckweed (DW). Shoot length (SL), root length (RL), panicle length (PL), SPAD, grain yield g pot^‒1^ (GY), no. of filled grains panicle^‒1^ (NFG), no. of unfilled grains panicle^‒1^ (NUG), 1000- grain weight (1000-GW), above-ground biomass (AGB), below-ground biomass (BGB), root-As (R-As), straw-As (S-As), grain-As (G-As), hydrogen peroxide (H_2_O_2_), malondialdehyde (MDA), activities of superoxide dismutase (SOD), catalase (CAT), ascorbate peroxidase (APX), BCF root (BCF-R), BCF straw (BCF-S), BCF grain (BCF-G), TF root-straw (TFr-s), TF root-grain (TFr-g) and soil-pH (pH)

We employed a response surface methodology to determine the optimal application doses of BC, VC, and DW to maximize GY and minimize G-As in *O. sativa* (Fig. 7). We maintained G-As at their minimum level while maximizing GY. Other parameters were kept within their ranges during analysis (Fig. 7). Following these conditions, the software generated 43 solutions. Among them, solution no. 1, with a desirability score of 0.976, was selected (Fig. 7). Ultimately, we found that utilizing BC (0.76%), VC (4.62%), and DW (290.0 g m^‒2^) led to higher GY (32.96 g pot^‒1^) and lower G-As content (0.189 mg kg^‒1^¹) in *O. sativa* (Fig. 7).

## Discussion

Arsenic (As) pollution poses a critical environmental and public health concern in Bangladesh, particularly due to the widespread contamination of groundwater. The extensive use of contaminated groundwater for irrigation exacerbates the issue because As accumulates in the soil and affects crops, notably *O. sativa*. The *O. sativa* has a propensity for absorbing As from both soil and water, making it susceptible to contamination. Given Bangladesh’s heavy reliance on *O. sativa* production for sustenance, this contamination presents a significant risk to public health. The long-term consumption of As-contaminated *O. sativa* can lead to serious health problems [43]. Furthermore, As inhibits the biomass growth and yield of *O. sativa*, compounding the agricultural and health challenges [11]. To address these issues, the present study was undertaken to investigate the effects of different combinations of BC, VC, and DW in improving the physio-morphological growth and grain yield of *O. sativa* while reducing the concentration of As in the grain.

The study found that the addition of BC, VC, and DW significantly improved the morphological growth parameters of *O. sativa*, such as plant height, root length, and panicle length (Fig. 1). The BC can modify soil physio-chemical properties [44], increase nutrient and water retention [45], inhibit harmful bacteria, absorb metal ions and pesticides, and increase soil pH, nutritional status, and cationic exchange capacity (CEC) [46]. The VC also plays a vital role in enhancing *O. sativa* growth due to increased nutrient availability in the soil [47]. Previous studies have shown that earthworms from VC enhance nitrogen levels and increase nutrient access to plants, promoting vegetative growth [48, 49].

In addition, we noticed that the combination of BC and VC considerably increased the soil pH (Table 4). The increase in pH can have several beneficial impacts on soil chemistry and plant growth. Elevated soil pH levels can reduce the solubility and movement of harmful substances such as As by facilitating the creation of less soluble arsenate compounds [50]. The alteration in pH might reduce the availability and absorption of As by plants, as evidenced by a decrease in As levels in the roots, straw, and grain of *O. sativa* with all amendment treatments in comparison to those without any amendments (Table 4; Figs. 3a-c and 5a-f). Moreover, the decrease in As uptake by plants following BC and VC treatments, suggesting a shift in the redox status of As within the soil-plant ecosystem towards less bioavailable forms [51]. This reduction can be attributed to factors such as the creation of reducing conditions, adsorption of As onto treatment materials, soil health, microbial activity enhancement, and complexation of As with organic matter present in VC [52]. Furthermore, the elevated pH caused by the addition of BC and VC may result in the formation of insoluble compounds, causing As to precipitate and making it less accessible for plants to absorb [53]. The PCA and heatmap also illustrate a negative association between high BC and VC content and As levels in *O. sativa*. Moreover, DW is known for absorbing many pollutants, including As. The DW in As-contaminated water absorbs and reduces As, which may improve *O. sativa* growing in As-contaminated soil [54].

The observed increase in SPAD values following the incorporation of BC and VC signifies a positive influence on enhancing chlorophyll content in *O. sativa* grown in As-contaminated soil. This enhancement can be attributed to several mechanisms associated with BC and VC amendments. Firstly, BC and VC can influence soil pH and ion exchange processes, with BC potentially increasing soil pH while VC buffers pH fluctuations. Optimal soil pH is crucial for chlorophyll synthesis as it affects the availability of essential nutrients like nitrogen and magnesium. Additionally, the high CEC of BC enhances nutrient retention and availability, further supporting chlorophyll production [55], as we noticed in the present study. This aligns with findings from previous studies that have demonstrated the role of compost and BC in increasing physiological activity and total chlorophyll concentrations in leaves [56]. The interactive effects of BC, VC, and DW on morphological growth and chlorophyll content in *O. sativa* were further supported by PCA analyses, heatmap, and interaction table, providing comprehensive evidence of their synergistic benefits (Fig. 6 and Table S1).

The interaction effect of BC, VC, and DW exhibited that the grain yield of *O. sativa* steadily enhanced with the increase of BC and VC levels in As-contaminated soil, regardless of DW concentrations (Fig. 4a-f). The PCA analysis, heatmaps, and regression coefficient values also showed that high dosages of BC and VC-treated *O. sativa* seedlings increased grain yield and yield-contributing features. The improved photosynthesis was linked to the increased production of *O. sativa* grain under As stress [57]. The present research showed that BC, VC, and DW applications substantially increased the SPAD value of chlorophyll content, which plays a significant role in improving the net photosynthetic rate (Fig. 1d). Considering the various nutrients in BC, VC, and DW, we assumed that nutrients such as N, P, K, Fe, Mn, Cu, and Mg from these organic fertilizers uptaken by *O. sativa* could improve photosynthesis and consequently enhance grain yield. The results of our research are consistent with earlier studies that have demonstrated that the administration of organic fertilizer at appropriate quantities can increase the levels of pigments and improve the yield of different plants under As stress [58, 59].

The As induces oxidative damage in plant leaves, as indicated by elevated levels of MDA and increased production of reactive oxygen species (ROS), including H_2_O_2_ [60]. In comparison to the control treatment, all combinations of BC, VC, and DW treatments significantly reduced H_2_O_2_ and MDA levels in *O. sativa* seedling leaves (Fig. 3d-e). Oxidative stress in plants, leading to irreversible damage to membrane structures through lipid peroxidation, is associated with the presence of MDA and H_2_O_2_ [33, 38]. Shabbir et al. [61] demonstrated that arsenic significantly exacerbates oxidative stress, resulting in substantial damage to membrane structures and cell death in plants. However, the application of BC and other organic fertilizers led to a notable decrease in H_2_O_2_ and MDA levels, thereby enhancing membrane stability. According to Siddiqui et al. [62], H_2_O_2_ poses greater harm when converted into highly toxic hydroxyl anions. Therefore, it is essential to convert these ROS into non-toxic compounds to support plant survival [43]. This conversion process is facilitated by numerous antioxidant enzymes found in different cellular compartments [63, 64].

The BC, VC, and DW have demonstrated significant potential in alleviating the negative effects of As-contamination on *O. sativa* seedlings, particularly through modulation of antioxidant enzyme activities. The BC application has been shown to enhance activities of key antioxidant enzymes such as SOD, CAT, and APX in the leaves of *O. sativa* seedlings grown in As-contaminated soils, aiding in the scavenging of ROS and reducing oxidative damage [61, 65]. Similarly, VC, rich in organic matter and beneficial microorganisms, can enhance antioxidant enzyme activities in rice seedlings under As stress, contributing to ROS detoxification and cellular protection [57]. This suggests that increased antioxidant enzyme activity could serve as a response mechanism to enhance As stress tolerance in plants. Antioxidant enzymes are overproduced under As stress to reduce ROS to non-toxic levels [42]. Enhanced antioxidant enzyme activities remove excess ROS, accelerate the immune system, and decrease the negative effect of stress conditions on different plant species [32, 66]. Findings of the current study from PCA, heatmap, and regression coefficient value also indicated that H_2_O_2_ and MDA contents and the activities of SOD, CAT, and APX in the *O. sativa* seedlings are positively correlated with the high BC-VC-containing treatments and negatively correlated with the low BC-VC containing treatments.

The bioconcentration factor (BCF) and translocation factor (TF) values are crucial indicators of the uptake and movement of metals by plants [67]. Understanding these values is essential for assessing the status and dynamics of As transfer from soil to plants [68]. Our experimental data revealed a decrease in BCF values for roots, straw, and grain as the levels of BC, VC, and DW increased, compared to the control (Table 4). The application of BC and VC likely contributed to a significant reduction in metal availability, leading to lower BCF values [68]. This reduction in metal accumulation is attributed to the ability of BC and VC to immobilize metals, thus decreasing their concentrations in plant tissues by enhancing overall biomass [69]. Similarly, the TF (TFr-s and TFr-g) consistently decreased with increasing incorporation of BC, VC, and DW. This indicates that the use of these amendments resulted in the immobilization of As in roots, thereby hindering its subsequent uptake and translocation to straw and grain. Furthermore, when BC, VC, and DW are employed in cultivating *O. sativa* in As-contaminated soil, several changes in soil parameters are expected. The BC aids in reducing As absorption by *O. sativa* plants through As adsorption, potentially lowering post-harvest soil As levels [70]. The VC enhances soil fertility by supplying essential plant nutrients, improving soil structure, and promoting microbial activity, which can aid in immobilizing and degrading As in post-harvest soil [25]. Additionally, DW, known for its ability to absorb heavy metals, could have sequestered As from the soil, thereby reducing As levels in post-harvest soil. Together, these amendments positively influence soil parameters by increasing nutrient availability, stimulating microbial activity, improving soil structure, and potentially mitigating As contamination. This creates a conducive environment for crop growth while minimizing the risk of As accumulation in *O. sativa* seedlings.

The primary aim of this study is to determine the optimal combination of BC, VC, and DW that can effectively reduce the As content in *O. sativa* grains while maintaining grain yield. Our findings indicate that under the conditions of BC (0.76%), VC (4.62%), and DW (290.0 g m^− 2^), we achieved the desired outcome, with the As content in *O. sativa* grains measuring 0.189 mg kg^− 1^ (Fig. 7). This level falls below the maximum inorganic As level permitted in husked *O. sativa*, as established by the Codex Alimentarius [71]. Given its potential practical implications, we suggest that the combined application of the aforementioned optimal doses of BC, VC, and DW could be recommended for *O. sativa* cultivation in As-contaminated areas. This approach holds promise for reducing As accumulation in *O. sativa* grains, thereby enhancing food quality and safety. However, additional field experiments are required to validate these results before making any recommendations, ensuring the findings’ reliability and reproducibility across diverse agricultural settings.

## Conclusions

Our study highlights the significant potential of BC, VC, and DW in mitigating As contamination effects on *O. sativa* cultivation. We have demonstrated their efficacy in enhancing key agronomic parameters, such as plant height, panicle length, and SPAD value, leading to a substantial increase in grain yield per pot. Moreover, these amendments show promise in reducing the detrimental effects of As, as evidenced by improvements in parameters like the number of filled grain panicles per plant and 1000-grain weight, ultimately addressing a critical concern in As-contaminated agricultural soils. Additionally, our investigation reveals a reduction in oxidative stress, indicated by decreased levels of H_2_O_2_ and MDA in leaves, also the As content in root, straw and grain. Utilizing RSM, we have identified an optimal composition of BC, VC, and DW that maximizes grain yield while minimizing As concentration in *O. sativa* grains. However, it’s important to acknowledge some limitations in our study, such as the small-scale experimental setup and the need for additional validation in field conditions. Future research should concentrate on understanding underlying mechanisms, performing long-term field experiments, and investigating synergistic effects with various amendments to improve *O. sativa* cultivation in As-contaminated soils. Overall, our research contributes to the current understanding and provides a basis for developing safer agricultural practices in areas affected by As and other heavy metals.

### Electronic supplementary material

Below is the link to the electronic supplementary material.

Supplementary Material 1

## Data Availability

All data generated or analysed during this study are included in this published article.

## Abbreviations

AS: Arsenic
BC: Biochar
VC: Vermicompost
DW: Duckweed
*O. sativa*: *Oryza sativa*
GY: Grain Yield
G-As: Grain As
RSM: Response Surface Methodology
CCD: Central Composite Design
SL: Shoot Length
RL: Root Length
PL: Panicle Length
NFG: Number of filled grains panicle^− 1^
NUG: Number of unfilled grains panicle^− 1^
1000-GW: 1000-grain weight
ABG: Above-ground Biomass
BGB: Below-ground Biomass
SPAD: Soil Plant Analysis Development
H_2_O_2_: Hydrogen Peroxide
MDA: Malondialdehyde
SOD: Superoxide Dismutase
CAT: Catalase
APX: Ascorbate Peroxidase
BCF: Bioconcentration Factor
TF: Translocation Factor
BCF-R: BCF Root
BCF-S: BCF Straw
BCF-G: BCF Grain
TFr-s: TF root-straw
TFr-g: TF root-grain
R-As: Root As
S-As: Straw As
PCA: Principal component analysis
ROS: Reactive oxygen species
