# Supplementary material for: Optimizing biochar, vermicompost, and duckweed amendments to mitigate arsenic uptake and accumulation in rice (Oryza sativa L.) cultivated on arsenic-contaminated soil
Source: BMC Plant Biol. 2024 Jun 13;24:545. doi: 10.1186/s12870-024-05219-w (PMC11177396; doi:10.1186/s12870-024-05219-w)
Supplement: Supplementary file 1 — Supplementary Material 1 [file 12870_2024_5219_MOESM1_ESM.docx]

**Supplementary materials**


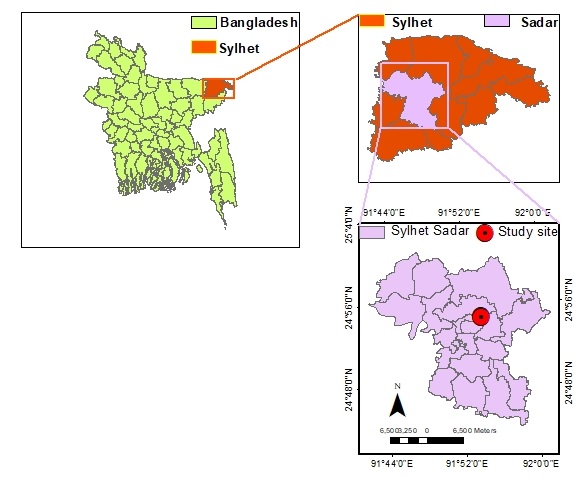


**Fig. S1.** Geographical location of the pot experiment.

**Fig. S2.** Graphic illustration of three factors and five levels central composite design.

**Fig. S3.** Depicts the effects of various biochar-vermicompost-duckweed (BC-VC-DW) regimes on phenotypic expression of *O. sativa* seedlings.

**Table S1.** Parameter coefﬁcients of the regression equation (Y= β_0_ + β_1_A + β_2_B + β_3_C + β_12_AB + β_13_AC + β_23_BC + β_11_A^2^ + β_22_B^2^ + β_33_C^2^) for shoot length (SL), root length (RL), panicle length (PL), SPAD, grain yield g pot^‒1^ (GY), No. of filled grains panicle^‒1^ (NFG), No. of unfilled grains panicle^‒1^ (NUG), 1000- grain weight (1000-GW), above ground biomass (AGB), below ground biomass (BGB), root-Arsenic (R-As), straw-Arsenic (S-As), grain-Arsenic (G-As), hydrogen peroxide (H_2_O_2_), malondialdehyde (MDA), activities of superoxide dismutase (SOD), catalase (CAT), ascorbate peroxidase (APX), bioconcentration factor of root (BCF-R), BCF-straw (BCF-S), BCF-grain (BCF-G), translocation factor root to straw (TFr-s), TF root to grain (TFr-g) and soil pH (pH).

| **Y** | **β_0_** | **β_1_** | **β_2_** | **β_3_** | **β_12_** | **β_13_** | **β_23_** | **β_1_²** | **β_2_²** | **β_3_²** |
| --- | --- | --- | --- | --- | --- | --- | --- | --- | --- | --- |
| **SL** | 130.32 | 2.88*** | 0.86 ^ns^ | 0.77 ^ns^ | -0.58 ^ns^ | 1.17 ^ns^ | 5.63*** |  |  |  |
| **RL** | 40.51 | 3.26*** | 0.1 ^ns^ | 0.03 ^ns^ | -2.69*** | -0.46 ^ns^ | 4.34*** |  |  |  |
| **PL** | 19.89 | 1.46*** | 0.49*** | 0.43*** |  |  |  |  |  |  |
| **SPAD** | 46.2 | 0.57*** | 1.41*** | -0.02 ^ns^ |  |  |  |  |  |  |
| **GY** | 30.55 | 1.07*** | 1.82*** | -0.24 ^ns^ | -0.36 ^ns^ | -0.01 ^ns^ | -0.02 ^ns^ | -0.16 ^ns^ | -0.08 ^ns^ | -0.67** |
| **NFG** | 73.76 | 0.62*** | 1.24*** | 0.68*** | 0.19 ^ns^ | 0.19 ^ns^ | 0.29 ^ns^ | -0.31* | -0.31* | -0.31* |
| **NUG** | 9.2 | 0.04 ^ns^ | 0.45* | -0.47* |  |  |  |  |  |  |
| **1000-GY** | 23.23 | 1.02*** | 0.32** | 0.22* |  |  |  |  |  |  |
| **AGB** | 40.46 | 1.06*** | 1.32*** | -0.84*** | -0.03 ^ns^ | 0.21** | -0.78*** |  |  |  |
| **BGB** | 19.03 | 2.13*** | 1.39*** | -1.71*** | 0.01 ^ns^ | -0.01 ^ns^ | 0.01 ^ns^ | 0.23* | 0.41*** | 0.4*** |
| **R-As** | 20.64 | -2.51*** | -1.28* | -1.37** |  |  |  |  |  |  |
| **S-As** | 1.94 | -0.16*** | -0.11*** | -0.11*** | -0.01^ns^ | 0.03^ns^ | 0.01^ns^ | 0.07** | 0.07** | 0.11*** |
| **G-As** | 0.029 | -0.004*** | -0.003*** | -0.004*** | 0.000 ^ns^ | -0.001 ^ns^ | 0.001 ^ns^ | 0.002*** | 0.002*** | 0.002*** |
| **H_2_O_2_** | 0.85 | -0.15*** | -0.11*** | -0.17*** | -0.01 ^ns^ | -0.01 ^ns^ | -0.01 ^ns^ | 0.09*** | 0.14*** | 0.07** |
| **MDA** | 5.3 | -0.5*** | -0.43*** | -0.63*** | 0.01 ^ns^ | 0.01 ^ns^ | 0.01 ^ns^ | 0.17*** | 0.19*** | 0.18*** |
| **SOD** | 23.5 | -3.35*** | -2.82*** | -4.27*** | -0.88*** | -0.08 ^ns^ | 0.25** | 2.04*** | 1.51*** | 2.21*** |
| **CAT** | 41.59 | -4.84*** | -3.29** | -3.78*** | -4.4** | 4.9*** | 0.35 ^ns^ | 2.78** | 3.32** | 2.07* |
| **APX** | 2.43 | -0.7*** | -0.42*** | -0.95*** | -0.04^ns^ | 0.17 ^ns^ | 0.08 ^ns^ | 0.51*** | 0.53*** | 0.56*** |
| **BCF-R** | 1.03 | -0.125*** | -0.064* | -0.068** |  |  |  |  |  |  |
| **BCF-S** | 0.097 | -0.008*** | -0.005*** | -0.005*** | -0.0004^ns^ | 0.001^ns^ | 0.0003^ns^ | 0.004** | 0.004** | 0.005*** |
| **BCF-G** | 0.01 | -0.001*** | -0.001*** | -0.001*** | -0.0001 | -0.0002^ns^ | 0.0002^ns^ | 0.001*** | 0.001*** | 0.001*** |
| **TFr-s** | 0.104 | 0.005* | 0.002 ^ns^ | 0.002^ns^ |  |  |  |  |  |  |
| **TFr-g** | 0.011 | 0.0002^ns^ | -0.0001^ns^ | -0.0004^ns^ | 0.0002^ns^ | -0.0001^ns^ | 0.00002^ns^ | 0.0002^ns^ | 0.001* | 0.0004^ns^ |
| **pH** | 6.15 | 0.45*** | 0.11*** | -0.39^ns^ |  |  |  |  |  |  |

ns = not significant, **p <* 0.05, ***p <* 0.01, ****p <* 0.001 indicate ANOVA significance.

**Table S2.** Statistical parameters from the analysis of variance for the regression models of different growth parameters of rice. Shoot length (SL), root length (RL), panicle length (PL), SPAD, grain yield g pot^‒1^ (GY), No. of filled grains panicle^‒1^ (NFG), No. of unfilled grains panicle^‒1^ (NUG), 1000- grain weight (1000-GW), above ground biomass (AGB), below ground biomass (BGB), root-Arsenic (R-As), straw-Arsenic (S-As), grain-Arsenic (G-As), hydrogen peroxide (H_2_O_2_), malondialdehyde (MDA), activities of superoxide dismutase (SOD), catalase (CAT), ascorbate peroxidase (APX), bioconcentration factor of root (BCF-R), BCF-straw (BCF-S), BCF-grain (BCF-G), translocation factor root to straw (TFr-s), TF root to grain (TFr-g) and soil pH (pH).

| **Growth parameters** | **Source** | **F-value** | **Sequential p-value** | **Lack of Fit p-value** | **R²** | **Adjusted R²** | **Predicted R²** | **Adequate Precision** | **C.V. %** | **Remarks** |
| --- | --- | --- | --- | --- | --- | --- | --- | --- | --- | --- |
| **SL** | 2FI | 23.75 | < 0.0001 | 0.21 | 0.93 | 0.9 | 0.59 | 19.65 | 1.28 | Suggested |
| **RL** | 2FI | 87.89 | < 0.0001 | 0.1 | 0.98 | 0.97 | 0.92 | 38.95 | 2.03 | Suggested |
| **PL** | Linear | 175.83 | < 0.0001 | 0.26 | 0.98 | 0.97 | 0.96 | 39.4 | 1.3 | Suggested |
| **SPAD** | Linear | 48.17 | < 0.0001 | 0.05 | 0.92 | 0.9 | 0.84 | 20.91 | 1.02 | Suggested |
| **GY** | Quadratic | 35.31 | < 0.0001 | 0.08 | 0.98 | 0.95 | 0.84 | 20.33 | 1.55 | Suggested |
| **NFG** | Quadratic | 24.66 | 0.0002 | 0.16 | 0.97 | 0.93 | 0.78 | 17.02 | 0.55 | Suggested |
| **NUG** | Linear | 3.89 | 0.0348 | 0.81 | 0.47 | 0.35 | 0.11 | 5.64 | 7.69 | Suggested |
| **1000-GW** | Linear | 41.68 | < 0.0001 | 0.28 | 0.91 | 0.88 | 0.83 | 19.59 | 1.56 | Suggested |
| **AGB** | 2FI | 308.05 | < 0.0001 | 0.27 | 0.99 | 0.99 | 0.97 | 58.71 | 0.42 | Suggested |
| **BGB** | Quadratic | 235.73 | < 0.0001 | 0.41 | 0.99 | 0.99 | 0.98 | 54.84 | 1.25 | Suggested |
| **R-As** | Linear | 17.23 | < 0.0001 | 0.048 | 0.79 | 0.75 | 0.66 | 13.21 | 7.8 | Suggested |
| **S-As** | Quadratic | 21.46 | 0.0003 | 0.04 | 0.96 | 0.92 | 0.73 | 14.89 | 3.05 | Suggested |
| **G-As** | Quadratic | 65.23 | < 0.0001 | 0.09 | 0.99 | 0.97 | 0.91 | 26.13 | 2.88 | Suggested |
| **H_2_O_2_** | Quadratic | 45.65 | < 0.0001 | 0.09 | 0.98 | 0.96 | 0.88 | 21.35 | 4.87 | Suggested |
| **MDA** | Quadratic | 187.95 | < 0.0001 | 0.46 | 0.99 | 0.99 | 0.97 | 48.17 | 1.47 | Suggested |
| **SOD** | Quadratic | 1855.33 | < 0.0001 | 0.09 | 0.99 | 0.99 | 0.99 | 143.59 | 0.67 | Suggested |
| **CAT** | Quadratic | 24.24 | 0.0002 | 0.06 | 0.97 | 0.93 | 0.76 | 15.85 | 4.83 | Suggested |
| **APX** | Quadratic | 46.65 | < 0.0001 | 0.05 | 0.98 | 0.96 | 0.87 | 20.99 | 6.92 | Suggested |
| **BCF-R** | Linear | 17.23 | < 0.0001 | 0.05 | 0.80 | 0.75 | 0.65 | 13.21 | 7.8 | Suggested |
| **BCF-S** | Quadratic | 21.46 | 0.0003 | 0.04 | 0.97 | 0.92 | 0.74 | 14.89 | 3.05 | Suggested |
| **BCF-G** | Quadratic | 65.23 | < 0.0001 | 0.09 | 0.99 | 0.97 | 0.91 | 26.13 | 2.88 | Suggested |
| **TFr-s** | Linear | 2.59 | 0.0974 | 0.115 | 0.37 | 0.22 | 0.15 | 4.9 | 7.08 | Suggested |
| **TFr-g** | Quadratic | 1.91 | 0.21 | 0.01 | 0.71 | 0.33 | 0.26 | 3.7 | 6.2 | Suggested |
| **pH** | Linear | 103.24 | < 0.0001 | 0.034 | 0.96 | 0.95 | 0.93 | 31.92 | 1.6 | Suggested |
